# Decoupling between calorimetric and dynamical glass transitions in high-entropy metallic glasses

**DOI:** 10.1038/s41467-021-24093-w

**Published:** 2021-06-22

**Authors:** Jing Jiang, Zhen Lu, Jie Shen, Takeshi Wada, Hidemi Kato, Mingwei Chen

**Affiliations:** 1 Institute for Materials Research, Tohoku University, Sendai, Japan; 2 Advanced Institute for Materials Research, Tohoku University, Sendai, Japan; 3 Institute of Physics, Chinese Academy of Sciences, Beijing, China; 4 School of Physical Sciences, University of Chinese Academy of Sciences, Beijing, China; 5 Department of Materials Science and Engineering, Johns Hopkins University, Baltimore, MD USA

**Keywords:** Structure of solids and liquids, Thermodynamics

## Abstract

Glass transition is one of the unresolved critical issues in solid-state physics and materials science, during which a viscous liquid is frozen into a solid or structurally arrested state. On account of the uniform arrested mechanism, the calorimetric glass transition temperature (*T*_*g*_) always follows the same trend as the dynamical glass transition (or *α*-relaxation) temperature (*T*_*α*_) determined by dynamic mechanical analysis (DMA). Here, we explored the correlations between the calorimetric and dynamical glass transitions of three prototypical high-entropy metallic glasses (HEMGs) systems. We found that the HEMGs present a depressed dynamical glass transition phenomenon, *i.e*., HEMGs with moderate calorimetric *T*_*g*_ represent the highest *T*_*α*_ and the maximum activation energy of *α*-relaxation. These decoupled glass transitions from thermal and mechanical measurements reveal the effect of high configurational entropy on the structure and dynamics of supercooled liquids and metallic glasses, which are associated with sluggish diffusion and decreased dynamic and spatial heterogeneities from high mixing entropy. The results have important implications in understanding the entropy effect on the structure and properties of metallic glasses for designing new materials with plenteous physical and mechanical performances.

## Abstract (web summary)

Here the authors study thermodynamic and dynamic glass transition of high entropy metallic glasses. Results show retarded α-relaxation and distinct crystallization resistance attributed to their sluggish diffusion and high-entropy mixing that is different from the traditional metallic glasses.

## Introduction

Metallic glass (MG), or, vitrified metal, is formed by rapidly quenching a liquid melt to bypass the crystallization process, and finally yields a configuration frozen state on a laboratory timescale^1–5^. Owing to the disordered atomic structure and out-of-equilibrium state, MGs exhibit unique and divergent thermodynamic and dynamic characteristics, especially when approaching the glass-transition temperature (*T*_*g*_)^3,6,7^. Conventionally, calorimetric *T*_*g*_ is defined as the temperature at which the specific heat has an abrupt jump and is commonly determined by calorimetric or thermal mechanical approaches^8–10^. From a dynamical relaxation perspective, diverse relaxation responses emerge with increasing temperature, from local reversible *β*-relaxation to global irreversible *α*-relaxation or a dynamic glass transition that is accompanied by the activation and subsequent percolation of shear transformation zones (STZs)^11–14^. Generally, since the consistency in the fundamental mechanism of glass transitions, the calorimetric *T*_*g*_ follows the same trend as that of the dynamic *α*-relaxation temperature (*T*_*α*_), i.e., MGs with high *T*_*g*_ always exhibit high *T*_*α*_^15–18^.

Conventionally, the traditional MGs design strategy is based on one principal element by adding secondary and more elements around deep eutectic points in the phase diagrams, which may restrict the discovery of numerous combinations with unique and divergent physical and mechanical properties^19–21^. Recently, an intriguing alloying approach was developed to design new type of near-equiatomic solid solution metallic alloys, defined as multiple-principal-element alloys or high-entropy alloys (HEAs), which broke the conventional metallurgy development strategy, and exhibited great potentials for developing advanced structural and functional materials^22–32^. Owing to the equimolar concentration of each component, HEAs have distinctive physical and mechanical properties^26–32^ arising from the effects of high configurational entropy, large lattice distortion, and sluggish diffusion as well as the cocktail effect^27,33^. Inheriting the distinct properties of MGs and HEAs, the new combinative glass-formed systems termed as “high entropy metallic glasses (HEMGs)” present high thermostability with depressed crystallization kinetics and superior magnetic properties, which reflect the theme of “more is different”^34–36^. However, the core critical behaviors of HEAs, especially the effect of sluggish diffusion on the thermodynamic and dynamic properties of HEMGs have not been systematically studied.

In this study, we utilized the strategy of the equivalent substitution elements to design La(Ce)-based, Pd(Pt)-based, and Ti(Zr)-based MGs and HEMGs, as the model systems to investigate the high-entropy effect on the structure and dynamics of glass-forming alloys.

## Results

### Decoupling of calorimetric *T*_*g*_ and dynamical *T*_*α*_

**Fig. 1:**
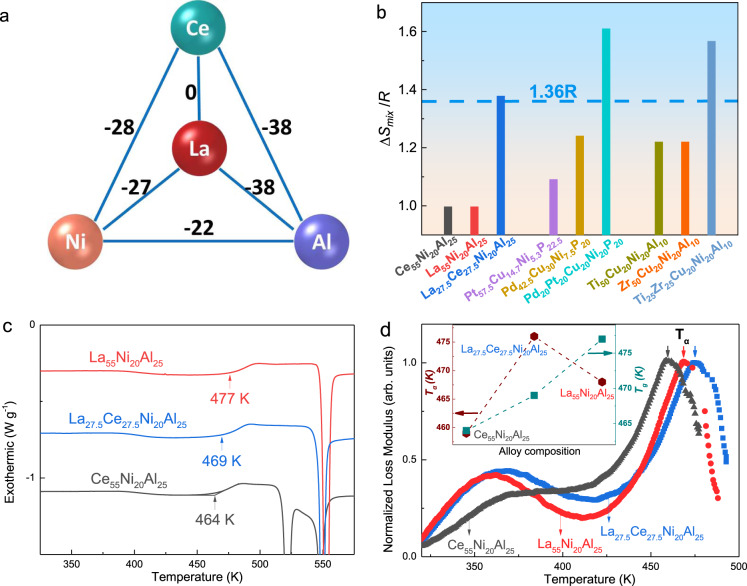
The mixing of entropy, calorimetric, and dynamical behaviors. **a** ∆*H* ^*mix*^ (kJ mol^−1^) for the constituting atomic pairs in the La(Ce)NiAl system. **b** Calculated entropies of mixing for the three MG systems. **c** DSC traces of the La(Ce)NiAl system with a heating rate of 0.33 K s^−1^. The arrows indicate the calorimetric glass-transition temperature (*T*_*g*_). **d** Temperature dependence of the loss modulus of the La(Ce)NiAl system at 1 Hz with a constant heating rate of 0.05 K s^−1^, normalized by the maximum peak value. The arrows indicate the *α*-relaxation temperature (*T*_*α*_), and the inset shows the evolution of *T*_*g*_ and *T*_*α*_ with the components.

Three types of prototypical MGs, viz. La_55_Ni_20_Al_25_ (Ce_55_Ni_20_Al_25_), Pd_42.5_Cu_30_Ni_7.5_P_20_ (Pt_57.5_Cu_14.7_Ni_5.3_P_22.5_), and Zr_50_Cu_20_Ni_20_Al_10_ (Ti_50_Cu_20_Ni_20_Al_10_) with different relaxation behaviors were selected as model systems in this study. Correspondingly, La_27.5_Ce_27.5_Ni_20_Al_25_, Pd_20_Pt_20_Cu_20_Ni_20_P_20_, and Ti_25_Zr_25_Cu_20_Ni_20_Al_10_ HEMGs were obtained by the partial replacement of the primary elements in the MGs and are hereafter referred to as LaCe-HEMG, PdPt-HEMG, and TiZr-HEMG, respectively. Figure 1a and Supplementary Fig. 1 display the enthalpy of mixing $\triangle {H}_{{AB}}^{{mix}}$ (kJ mol^−1^) values of the different atomic pairs in these representative MGs systems^37^. The value of zero for $\triangle {H}_{{AB}}^{{mix}}$ of the La–Ce pair and similar values for La and Ce with Ni and Al suggest that elemental substitution causes negligible changes in terms of the chemical effect on the relaxation behaviors^38^. Figure 1b presents the entropy of mixing of the three MGs systems, calculated by $\triangle {S}_{{mix}}=-R{\sum}_{i=1}^{n}{x}_{i}{\rm{ln}}{x}_{i}$, where *R* is the gas constant and *x*_*i*_ is the mole fraction of the *i*th element^27^. The designed HEMGs yield the values of $\triangle {S}_{{mix}}$ with 1.38 *R*, 1.67 *R*, and 1.57 *R* for LaCe-HEMG, PdPt-HEMG, and TiZr-HEMG, respectively, which are higher than the value generally defined as high entropy (1.36 *R*)^33^. Differential scanning calorimeter (DSC) traces of La_55_Ni_20_Al_25_, Ce_55_Ni_20_Al_25_, and LaCe-HEMG obtained at a heating rate of 0.33 K s^−1^ are shown in Fig. 1c. The obvious glass transition and crystallization signals confirm the glassy state of the three samples. Calorimetric *T*_*g*_ is determined from the intersection of the tangent lines of the onset of transformation and is indicated by arrows (Fig. 1c). LaCe-HEMG with the highest $\triangle {S}_{{mix}}$ yields an intermediate *T*_*g*_, PdPt-HEMG and TiZr-HEMGs show similar tendencies (Supplementary Figs. 2a and 3a), which are consistent with the rule of mixture^39,40^. In general, MGs exhibit multicomplex relaxation dynamics and *α*-relaxation as the main relaxation mode is directly related to viscous flow and the glass transition^3,41^. Figure 1d presents the temperature dependence of the loss modulus (*G*^*”*^) of La_55_Ni_20_Al_25_, Ce_55_Ni_20_Al_25_, and LaCe-HEMG at 1 Hz with a constant heating rate of 0.05 K s^−1^ (normalized by the maximum peak value). Two relaxation modes are clearly exhibited, which are termed as the *β*-relaxation and *α-*relaxation, from the low to high temperatures, respectively. With half of La replaced by Ce, LaCe-HEMG exhibits the highest *α-*relaxation temperature (*T*_*α*_), which is not synchronous with the calorimetric *T*_*g*_ (Fig. 1c). According to conventional wisdom, a glass with a higher calorimetric *T*_*g*_ always poses higher difficulty in activating *α-*relaxation and thus has a larger value of *T*_*α*_^15,42^. Besides, *T*_*α*_ presents a monotonous tendency in the case of regular element substitution^15^ and follows the rule of mixture, which is the same as the calorimetric *T*_*g*_. Nevertheless, LaCe-HEMG, with a modest thermodynamic devitrification behavior, exhibits the uppermost dynamic glass-transition process (inset of Fig. 1d). Identical results were observed in the other two MGs systems, viz. Pd(Pt)CuNiP and Ti(Zr)CuNiAl (Supplementary Fig. 2b and Supplementary Fig. 3b). In addition, the consecutive decreased storage modulus excludes the possible crystallization near the *α*-relaxation process as shown in Supplementary Fig. 4, which indicates that the *α*-relaxation peak is not terminated by the early crystallization. Therefore, our findings indicate a decoupling between the calorimetric and dynamical glass transitions of HEMGs.

### Fragility and viscosity of HEMGs

**Fig. 2:**
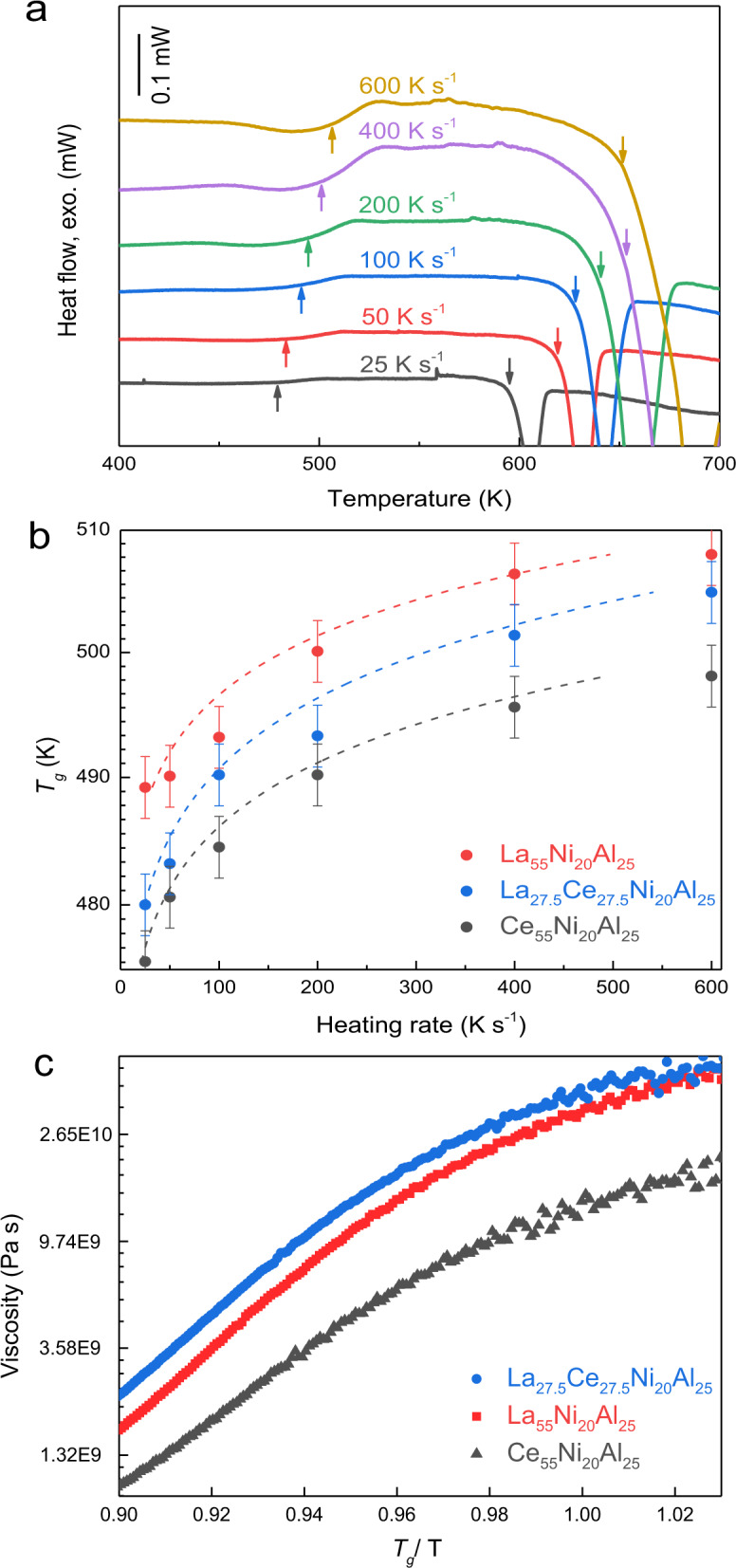
The fragility and viscosity behaviors of the La(Ce)NiAl system. **a** DSC traces of LaCe-HEMG with heating rates ranging from 25 to 600 K s^−1^. The left- and right-side arrows indicate *T*_*g*_ and *T*_*x*_, respectively. **b** Heating rate dependence of *T*_*g*_ for the La(Ce)NiAl MGs system and the corresponding VFT fitting traces (dashed lines). The error bars were obtained by standard deviation from three measurements of *T*_*g*_ on a DSC curve. **c** Nonequilibrium viscosity near the glass-transition region for the La(Ce)NiAl MGs, obtaining at a constant heating rate of 0.33 K s^−1^.

Fragility and viscosity are the most fundamental properties, reflecting the temperature-dependent relaxation and dynamic heterogeneity of glasses in supercooled liquid regions. Therefore, the divergent calorimetric and dynamical glass-transition behaviors of the HEMGs are expected to be disclosed by fully understanding the kinetic behaviors near the glass transition. Fragility can be determined by the variation in *T*_*g*_ as a function of heating rate^42,43^. Figure 2a shows the DSC traces with the heating rates ranging from 25 to 600 K s^−1^ for La(Ce)-HEMG system. *T*_*g*_ and the crystallization temperature (*T*_*x*_) shift to higher temperatures with the increase of heating rate, which arises from the involvement of thermal activation in these kinetic processes. Figure 2b presents the heating rate dependence of *T*_*g*_ for La_55_Ni_20_Al_25_, Ce_55_Ni_20_Al_25_, and LaCe-HEMG. The particular average structural relaxation time, *τ*, can be obtained by $\tau =\frac{\triangle {T}_{g}}{{q}_{H}}$, where ${\triangle T}_{g}={T}_{g}^{{end}}-{T}_{g}^{{onset}}$ is the width of the glass transition with a constant value of 30 K, and *q*_*H*_ is the cooling rate. Furthermore, *τ* can be well described by the Vogel–Fulcher–Tammann (VFT) equation^43^:

1 $$\tau ={\tau }_{{{\infty }}}{{\exp }}\left(\frac{D{T}_{0}}{T-{T}_{0}}\right),$$

where *τ*_*∞*_ is the relaxation time at infinite temperature with a constant value of 1 × 10^−14^ s, *T*_*0*_ is the VFT temperature, *D* is the fragility parameter, and fragility *m* = 16 + 590/*D*^44^. The *m* values for La_55_Ni_20_Al_25_, Ce_55_Ni_20_Al_25_, and LaCe-HEMG were calculated as 42, 39, and 35, respectively, by fitting the curves in Fig. 2b with Eq. (1). In comparison with other MGs, LaCe-HEMG exhibits a strong glass behavior in terms of the lowest fragility.

Figure 2c presents the nonequilibrium viscosity of the La(Ce)NiAl MGs as a function of temperature, which was measured by thermomechanical analysis (TMA). As the temperature increases above *T*_*g*_, the viscosity decreases rapidly. While, the viscosity of LaCe-HEMG is obviously higher than the others, suggesting the higher dynamic stability and more sluggish dynamic behavior of HEMG upon heating. Therefore, a plausible origin of the retarded *α*-relaxation for LaCe-HEMG could be sluggish diffusion from the high-entropy effect in HEAs^24,25^.

### Atomic structure

**Fig. 3:**
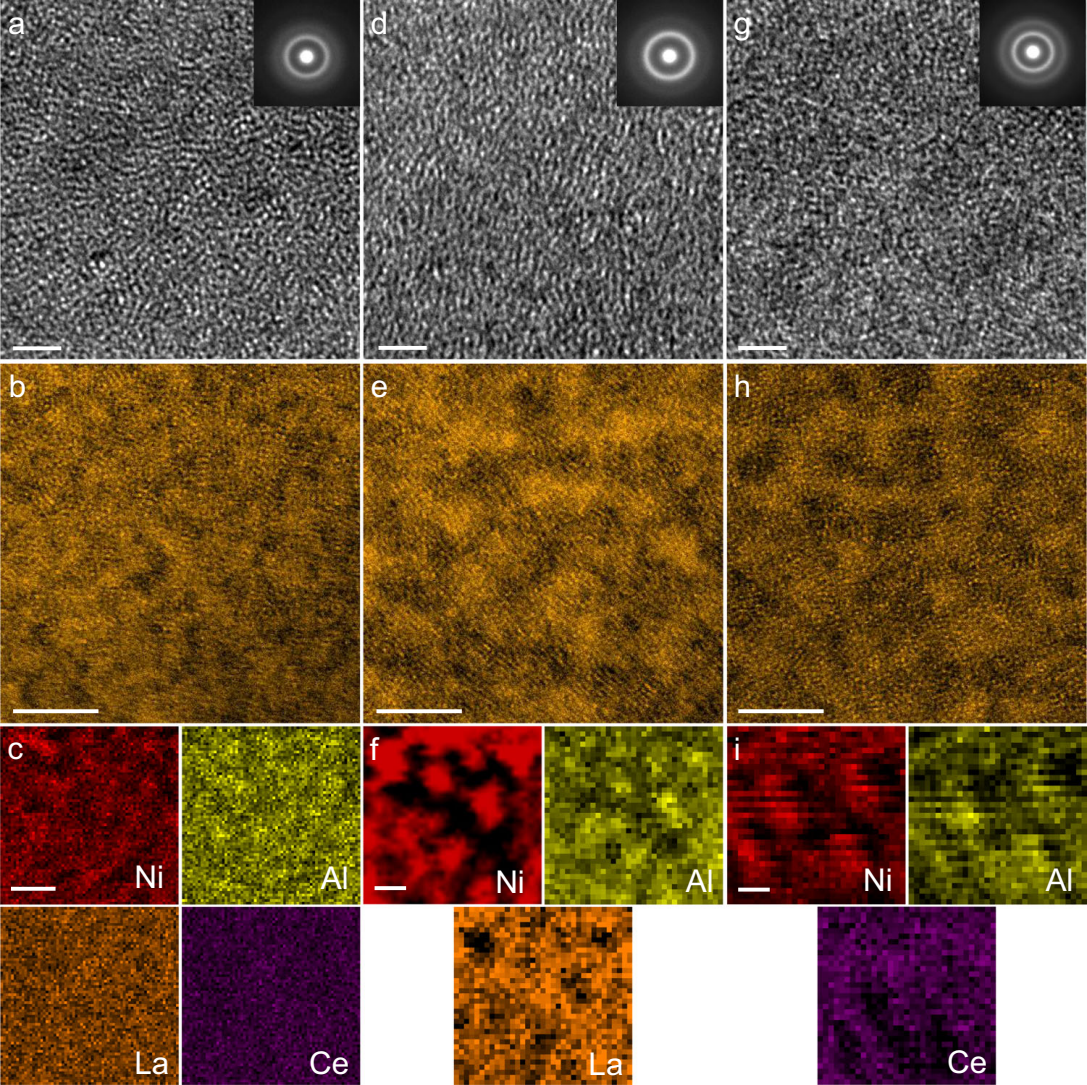
The atomic structures and corresponding elemental distributions of the La(Ce)NiAl system. **a**, **d**, **g** HRTEM images presenting uniform mazelike patterns for the as-cast LaCe-HEMG, La_55_Ni_20_Al_25_, and Ce_55_Ni_20_Al_25_, respectively, the corresponding SAED patterns (insets) demonstrating the typical amorphous structures. Scale bars: 2 nm. **b**, **e**, **h** HAADF-STEM images of LaCe-HEMG, La_55_Ni_20_Al_25_ and Ce_55_Ni_20_Al_25_, respectively. Scale bars: 5 nm. **c**, **f**, **i** Corresponding EDS chemical mappings for LaCe-HEMG, La_55_Ni_20_Al_25_ and Ce_55_Ni_20_Al_25_, respectively. LaCe-HEMG exhibits relatively homogeneous elemental distributions. Scale bars: 5 nm.

The relaxation behaviors of MGs are related to their spatial heterogeneity in the nanoscale domains^12,45^. Fig. 3a, d, g presents high-resolution transmission electron microscope (HRTEM) images of LaCe-HEMG, La_55_Ni_20_Al_25_, and Ce_55_Ni_20_Al_25_, respectively. The homogeneous mazelike features and the diffraction halo in the selected area electron diffraction (SAED) patterns (inset in Fig. 3a, d, g) confirm the amorphous nature of the samples. Apparently, no clear atomic structural differences and phase separation could be identified in the three MGs down to the sub-nanoscale from the phase-contrast images. However, when scanning transmission electron microscopy (STEM) analysis with a high-angle annular dark-field (HAADF) detector was performed to image the local atomic structures (Fig. 3b, e, h), the HAADF-STEM images present obvious contrast variations with domain sizes of several nanometers. It appears that the contrast variation of LaCe-HEMG is slightly ambiguous, together with a concomitant decrease in the domain size. The averaged domain sizes, measured by a rotationally averaged fast Fourier transform method^46^, correspond to feature lengths of 2.491 ± 0.297 nm, 2.380 ± 0.248 nm, and 1.851 ± 0.206 nm for La_55_Ni_20_Al_25_, Ce_55_Ni_20_Al_25_, and LaCe-HEMG, respectively (Supplementary Table 1). Accordingly, LaCe-HEMG with the highest value of $\triangle {S}_{{mix}}$ possesses the least contrast variation and the smallest domain size. Since the HAADF contrast indicates local chemical or density variations, STEM energy-dispersive spectroscopy (EDS) with a spatial resolution of ~3 Å was applied to further reveal the elemental distributions. As shown in Fig. 3f, i, La_55_Ni_20_Al_25_ and Ce_55_Ni_20_Al_25_ present obvious heterogeneous chemical variations with the sizes of ~3 nm coinciding with the domain size. The dark regions in the HAADF images are enriched with Al and Ni while La or Ce separates from Al and Ni and generates the bright regions. However, La and Ce are evenly distributed in LaCe-HEMG with weeny elemental variations, while evident chemical fluctuations of Al and Ni are still visible, as shown in Fig. 3c. Therefore, the contrast in the HAADF images is attributable to the nanoscale chemical fluctuation. The higher *T*_*α*_ of HEMGs plausibly originates from these relatively inconspicuous chemical variations with less pronounced spatial contrast heterogeneity and sluggish diffusion, which may depress the dynamical relaxation behaviors near the glass-transition temperatures.

### Crystallization behavior

**Fig. 4:**
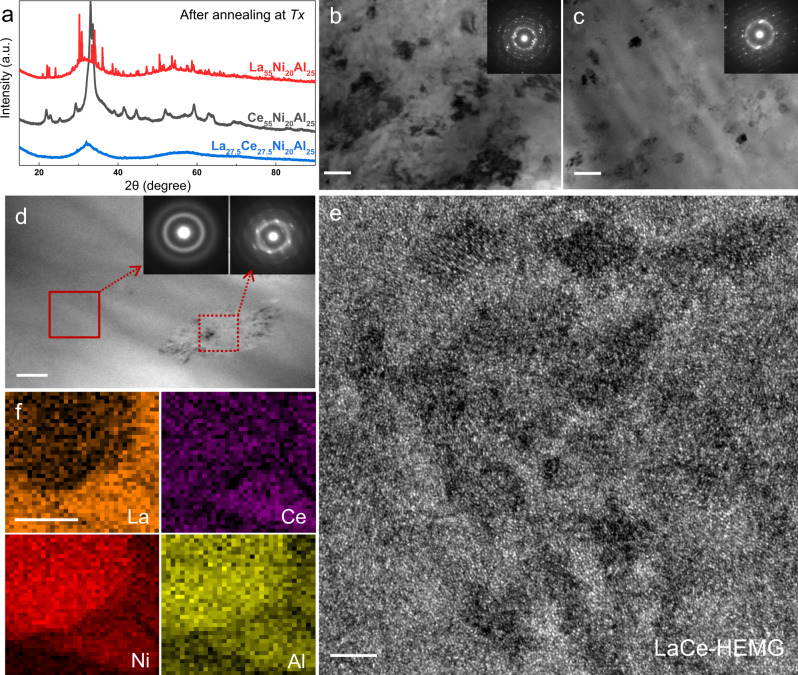
The crystallization behavior of the La(Ce)NiAl system. **a** XRD profiles of the La(Ce)NiAl system illustrating the depressed crystallization characterization of LaCe-HEMG. **b**, **c**, **d** Corresponding TEM images of crystallized La_55_Ni_20_Al_25_, Ce_55_Ni_20_Al_25_, and LaCe-HEMG, respectively. The insets show the respective SAED patterns. Scale bars: 100 nm. **e** HRTEM image of the crystallized region (dashed red frame in (**d**)) showing nanocrystals with an average size of 10 nm embedded in the amorphous matrix of LaCe-HEMG. Scale bar 5 nm. **f** EDS mappings of crystallized LaCe-HEMG. Scale bar: 50 nm.

HEMGs with higher *T*_*α*_ suggest sluggish cooperative atomic movement in the moderately supercooled liquid states, which, in principle, should also influence the crystallization behavior. Figure 4a shows the XRD spectra of crystallized La_55_Ni_20_Al_25_, Ce_55_Ni_20_Al_25_, and LaCe-HEMG by heating to *T*_*x*_ of each alloy at a constant rate of 0.33 K s^−1^ and immediately cooled to room temperature. Significantly Bragg peaks emerge in the XRD profiles of La_55_Ni_20_Al_25_ and Ce_55_Ni_20_Al_25_, indicating the appearance of obvious crystallization productions. In contrast, LaCe-HEMG still presents a broad amorphous halo superimposed with a few weak crystalline peaks, demonstrating that LaCe-HEMG is composed of a large portion of the amorphous phase and exhibits high resistance to crystallization. Figure 4b, c displays the corresponding TEM images of crystallized La_55_Ni_20_Al_25_ and Ce_55_Ni_20_Al_25_, respectively, in which crystalline grains are widely emerged from the amorphous matrix with the size around 100 nm. In contrast, LaCe-HEMG presents few precipitates with a size of hundreds of nanometers in the amorphous matrix (Fig. 4d), which appears to be formed by heterogeneous crystallization. In addition, the SAED patterns of the three heat-treated specimens still exhibit amorphous diffraction halos, suggesting the insufficient crystallization processes (the inset of Fig. 4b–d). Figure 4e shows the HRTEM image of the crystallization region for LaCe-HEMG (dashed red frame in Fig. 4d), the large precipitate is comprised of nanocrystals with an average grain size of ~8 ± 1.7 nm, which are embedded in the amorphous matrix and smaller than the crystallites in the crystallized La_55_Ni_20_Al_25_ and Ce_55_Ni_20_Al_25_ MGs (Supplementary Fig. 5 and Supplementary Table 1). It appears that HEMGs demonstrate a suppressed crystal nucleation and growth and, consequently, the enhanced thermal stability of the supercooled liquids.

## Discussion

**Fig. 5:**
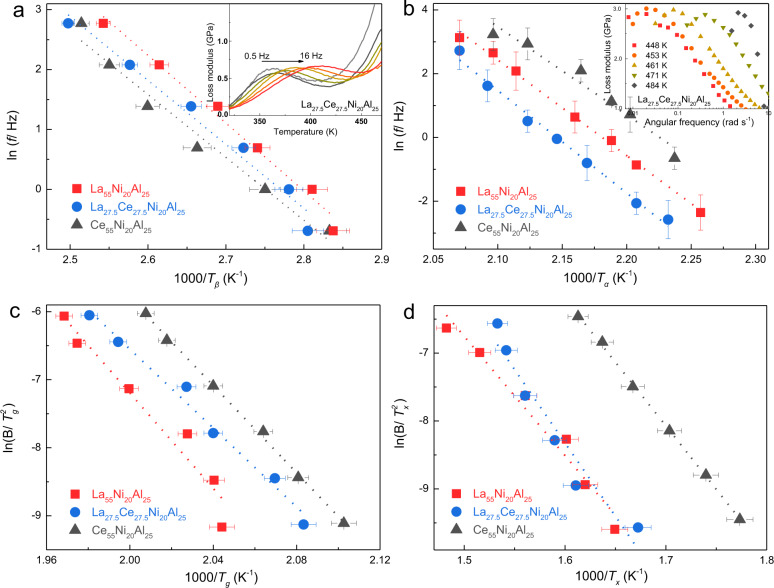
The activation energies for *β*-relaxation, *α*-relaxation, glass transition, and crystallization of the La(Ce)NiAl system. **a** Arrhenius plots of the temperature and frequency dependence of the peak temperature of *β-*relaxations for the La(Ce)NiAl system. The inset shows the temperature-dependent loss modulus of LaCe-HEMG with testing frequencies ranging from 0.5 to 16 Hz. **b** Frequency and temperature dependence of the peak temperature of *α*-relaxations fitted with the Arrhenius relationship. The inset presents the frequency-dependent loss modulus of LaCe-HEMG at different temperatures. **c**, **d** Kissinger plots of thermal *T*_*g*_ and *T*_*x*_ versus heating rate for the La(Ce)NiAl system, respectively. The error bars were obtained by standard deviation from three measurements of *T*_*β*_, *T*_*α*_, and *T*_*g*_, *T*_*x*_ on DMA and DSC curves, respectively.

HEMGs exhibit retarded *α-*relaxation (dynamical glass transition) and distinct crystallization resistance, which may originate from the sluggish diffusion hypothesis on account of the effect of high mixing entropy. *β*-relaxation (or Johari-Goldstein relaxation) is considered the dynamical precursor of *α*-relaxation and plays a promising role in understanding the dynamical glass transition^47,48^. The frequency dependence of *β*-relaxation evolution was explored by dynamic mechanical analysis (DMA) with an amplitude of 0.1% and testing frequencies (*f*) ranging from 0.5 to 16 Hz (inset of Fig. 5a and Supplementary Fig. 6). Figure 5a shows the frequency dependence of the *β*-relaxation peak temperatures (Supplementary Fig. 6 for details) for La_55_Ni_20_Al_25_, Ce_55_Ni_20_Al_25_, and LaCe-HEMG. The peaks were fitted with the Arrhenius relation^12^, $f={f}_{\infty }{{\exp }}(-{E}_{\beta }/{RT})$, where *f*_*∞*_ is the pre-factor and *E*_*β*_ is the activation energy of *β*-relaxation. The *E*_*β*_ values (Table 1) were calculated to be 85.8, 88.8, and 93.9 kJ mol^−1^ for Ce_55_Ni_20_Al_25_, LaCe-HEMG, and La_55_Ni_20_Al_25_, respectively. The *E*_*β*_ increases with Ce replaced by La, which agrees with previous reports and suggests that the activation of the *β*-relaxation mode follows a monotonous evolution according to the mean chemical affinity between the constituent elements^16,38^. LaCe-HEMG exhibits moderate activation energy for *β*-relaxation which follows the same tendency as calorimetric *T*_*g*_. Since *β*-relaxation only involves local atomic motion, the high-entropy effect appears not to influence short-range atomic diffusion. Accordingly, the temperature-dependence of *α*-relaxation is shown in inset of Fig. 5b and Supplementary Fig. 7. The activation energy values of *α*-relaxation (*E*_*α*_) (Fig. 5b and Table 1) were determined to be 229.8, 249.6, and 267.6 kJ mol^−1^ for Ce_55_Ni_20_Al_25_, La_55_Ni_20_Al_25_, and LaCe-HEMG from the Arrhenius plot, $f={f}_{\infty }{{\exp }}(-{E}_{\alpha }/{RT})$. Remarkably, LaCe-HEMG shows the highest *α*-relaxation activation energy, suggesting that HEMGs have the highest energy barrier for the dynamical glass transition, and thus the highest *T*_*α*_. Figure 5c, d shows the Kissinger plots of thermal *T*_*g*_ and *T*_*x*_ at different heating rates from the flash DSC traces (Fig. 2a). The measured activation energies for the calorimetric glass transition and crystallization are listed in Table 1. Again, LaCe-HEMG has intermediate activation energy for the glass transition and the highest energy for crystallization, which is in line with the calorimetric *T*_*g*_ and crystallization behavior.

**Table 1:** Activation energies of dynamic *β*-relaxation, *α*-relaxation, and calorimetric glassy transition, crystallization in the La(Ce)NiAl system.

Activation energy (kJ mol^−1^)	*E*_*β*_	*E*_*α*_	*E*_*g*_	*E*_*x*_
La_55_Ni_25_Al_20_	93.9 ± 4	249.6 ± 5	291.7 ± 5	146.1 ± 2
Ce_55_Ni_25_Al_20_	85.8 ± 4	229.8 ± 8	267.1 ± 5	155.6 ± 2
La_27.5_Ce_27.5_Ni_25_Al_20_	88.8 ± 4	267.6 ± 1	241.4 ± 5	178.5 ± 2

**Fig. 6:**
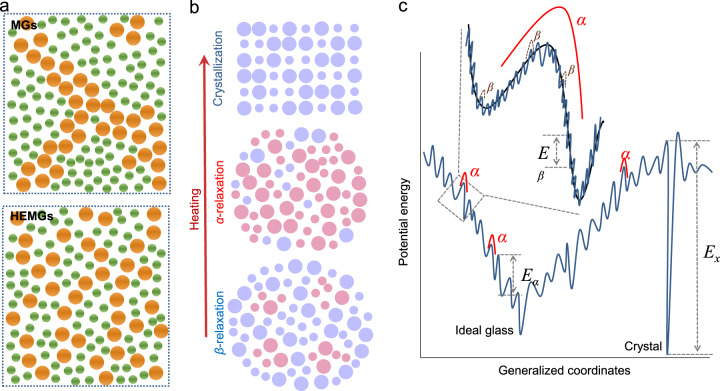
Schematic illustration of structure and energy landscape of MGs. **a** Microstructure illustration of HEMGs and traditional MGs. Based on the EDS results, HEMGs present relatively homogeneous elemental distributions which might originate from the high mixing entropy. **b** Correlation between the evolution of STZs and relaxation behaviors with increasing temperature. The MGs finally yield crystallization state at *T*_*x*_. **c** Schematic potential energy landscape for MGs, *β*-relaxation relates a reversible hopping event in an inherent megabasin, while *α*-relaxation corresponds to an irreversible hopping event across different landscape megabasins.

These structural characterization and dynamic measurements build up a comprehensive picture of the decoupling between the calorimetric *T*_*g*_ and DMA *T*_*α*_ of HEMGs as illustrated in Fig. 6. HEMGs with nearly equimolar components and high configuration entropy, could suppress the dynamic heterogeneity in the supercooled liquids on account of the reduced Gibbs free energy, and then inherit to the glassy state with relatively small and homogeneous nanoscale domains (Figs. 3b and 6a)^49^. These nanoscale spatial domains with an average size of ~2 nm are analogous to the size of STZs, and therefore might be associated with the structural origin of the *β*-relaxation process^14^ (Fig. 6b). Consequently, HEMGs with small nanoscale domains possess vibrant potential STZs on account of the favorable cooperative transition probability by “Adam-Gibbs” theory^50^, with the minor activation energy of *β*-relaxation (Fig. 5a and Table 1).

Furthermore, *β*-relaxation could be associated with the activation of isolated STZs, which are confined within the elastic matrix and do not involve long-range diffusion^14^. Therefore, the *β*-relaxation behavior of HEMGs is nearly unaffected by the sluggish diffusion effect. When the density and size of STZs reach a critical point with increasing temperature, their percolation of STZs contributes to distinct *α*-relaxation^51^. Figure 6b, c illustrates the relationship between *β*-relaxation and *α*-relaxation based on the structural and energy perspectives. In the *α*-relaxation process, the expansion and percolation correspond to an increased mismatch penalty between STZs, which relates to long-range cooperation rearrangement and atomic diffusion^52,53^ (Fig. 6b). Small STZs in HEMGs might expect to consume excess energy to reach a critical transition size^52,53^, and subsequent percolation with long-range propagation could plausibly be suppressed by sluggish diffusion of HEMGs. Therefore, HEMGs with relatively homogeneous structures (Figs. 3b and 6a) inhibit large-scale atomic migration in the process of *α*-relaxation, resulting in the delayed *α*-relaxation transition and elevated activation energy (Fig. 1d and Table 1). Analogously, the STEM-EDS mappings of crystallized MGs (Fig. 4f and Supplementary Fig. 5) verify a long-range diffusion and migration, suggesting that sluggish diffusion affects the crystallization behaviors of HEMGs and results in higher activation energy (Figs. 5d, 6c and Table 1).

Besides relaxation, the calorimetric glass transition is accompanied by an increase in heat capacity with the generation of free volumes near *T*_*g*_, which is not sensitive to the heterogeneous domain size^10^. Entropy might induce negligible effects on the thermal glass-transition behaviors, which is consistent with the fact that LaCe-HEMG presents the relatively low activation energy of calorimetric *T*_*g*_ (Fig. 5c and Table 1). Therefore, the calorimetric *T*_*g*_ could be mainly determined by local thermal diffusion and follow the rule of mixture characterized by a monotonous relationship with composition. Base on systematic examinations of the calorimetric and dynamical glass transitions and atomic structures, we can conclude that the high mixing entropy affects the glass transition and crystallization behaviors of HEMGs, as illustrated by the proposed energy landscape (Fig. 6c). It is expected that the HEA ‘core effect’, i.e., sluggish diffusion on the spatial heterogeneity and the decreased dynamics of supercooled liquids, will provide freedom to solve the fundamental issues, such as the glass transition, relaxations, and aging behaviors, of MGs.

## Methods

### Sample preparation

Master alloys with the nominal atomic percent compositions of La_55_Ni_20_Al_25_, Ce_55_Ni_20_Al_25_, La_27.5_Ce_27.5_Ni_20_Al_25_, Pd_42.5_Cu_30_Ni_7.5_P_20_, Pt_57.5_Cu_14.7_Ni_5.3_P_22.5_, Pd_20_Pt_20_Cu_20_Ni_20_P_20_, Zr_50_Cu_20_Ni_20_Al_10_, Ti_50_Cu_20_Ni_20_Al_10_, and Ti_25_Zr_25_Cu_20_Ni_20_Al_10_ were prepared by arc-melting mixtures of raw metals (purity >99.9 mass %) in an Ar atmosphere purified with a Ti getter. Glassy ribbons (thickness around 50 μm) were produced by melt-spinning onto a single Cu wheel at 3000 r.p.m. using Ar gas overpressure. La_55_Ni_20_Al_25_, Ce_55_Ni_20_Al_25_, and La_27.5_Ce_27.5_Ni_20_Al_25_ glass ribbons were carefully polished to 5 μm by 4000# sandpaper for preparing TEM specimens in advance. The final TEM specimens were prepared by ion milling (Gatan, PIPS II 695) with 3 kV and finally polished with low-voltage (0.5 kV) with a liquid nitrogen-cooled stage.

### Differential scanning calorimetry (DSC) measurements

The Perkin Elmer DSC 8500 was used to perform the thermodynamic transition process of the glassy ribbons with 20 mL min^-1^ flowing pure argon gas to prevent possible oxidation during heating. The values of glass-transition temperature *T*_*g*_ (Fig. 1) were determined using DSC at a constant heating rate of 0.33 K s^−1^. Flash DSC (Mettler, Flash DSC 1) with wide heating rates was used to measure the glass transition and crystallization behaviors of La_55_Ni_20_Al_25_, Ce_55_Ni_20_Al_25_, and La_27.5_Ce_27.5_Ni_20_Al_25_ samples with the heating rates range from 25 K s^−1^ to 600 K s^−1^. Tiny samples with dimensions of about 40 × 50 × 6 μm were cut from a melt-spun ribbon. The samples were then loaded on the center of the Flash DSC sensor chip under an optical microscope. The sensor and the sample were protected from oxygen and moisture under a steady Ar flow of 20 mL min^−1^ during the whole measurement.

### Dynamic mechanical analysis (DMA)

The RSA-G2 DMA (TA Instruments) was used to measure the loss and storage moduli of the MGs ribbons. The loss modulus shows a sharp maximum near the glass-transition temperature associated with *α*-relaxation. The broad maximum at lower temperature represents the *β*-relaxation. The *α*-relaxation temperature (*T*_*α*_, Fig. 1) was determined by heating the glassy ribbons from room temperature to over crystallization temperature at 1 Hz with a heating rate of 0.05 K s^−1^. The frequency dependence of *β*-relaxation evolution (Fig. 5) was explored by DMA with an amplitude of 0.1% and testing frequency *f* ranging from 0.5 to 16 Hz. The temperature dependence of *α-*relaxation evolution (Fig. 5) explored by DMA with an amplitude of 0.1% and testing temperature ranging near *T*_*g*_.

### Viscosity measurement

The thermomechanical analyzer Q400 TMA (TA Instruments) was used to measure the length change of glassy ribbon samples during heating in the argon fluxion atmosphere (20 mL min^−1^). The measurement of viscosity in Fig. 2 was done with a heating rate of 0.33 K s^−1^ from room temperature to crystallization temperature with a contrast probe load of 0.1 N. The value of viscosity (*η*) was calculated from the flow stress by the equation $\eta =\frac{\sigma }{3\dot{\varepsilon }}$, where $\sigma$ is the stress and $\dot{\varepsilon }$ is the strain rate.

### X-rays diffraction

The phase transformation after annealing the glassy ribbons was characterized by RIGAKU Ultima IV X-Ray diffractometer with monochromatic Cu K_α_ radiation. The ribbons were heated to the onset temperature of crystallization followed by cooling down.

### Transmission electron microscopy characterization

Microstructures of the specimens were characterized by a Cs-corrected TEM (JEM-2100F, JEOL, 200 kV) equipped with double spherical aberration correctors for both the probe-forming and image-forming lenses. The high-resolution TEM images were captured by a television-rate camera (Gatan, UltraScan 1000), and the high-angle annular dark-field (HAADF) images were recorded using an annular-type scanning TEM detector. Energy-dispersive spectroscopy (EDS) mappings were acquired at STEM mode with a scan step of 3 Å for elemental analysis.

## Supplementary information

Supplementary Information

## Data Availability

The data that support the findings of this study are available from the corresponding author upon reasonable request.
