# Supplementary material for: Decoupling between calorimetric and dynamical glass transitions in high-entropy metallic glasses
Source: Nat Commun. 2021 Jun 22;12:3843. doi: 10.1038/s41467-021-24093-w (PMC8219663; doi:10.1038/s41467-021-24093-w)
Supplement: Supplementary file 1 — Supplementary Information [file 41467_2021_24093_MOESM1_ESM.pdf]

Supplemental materials for

Decoupling between calorimetric and dynamical glass transitions in high-entropy metallic glasses

Jing Jiang, Zhen Lu, Jie Shen, Takeshi Wada, Hidemi Kato* & Mingwei Chen*

*Corresponding author. Email:

hikato@imr.tohoku.ac.jp (H. Kato) or mwchen@jhu.edu (M.W. Chen)

Supplemental materials including:

Supplementary Figures 1-7

Supplementary Table 1

Supplementary Figures

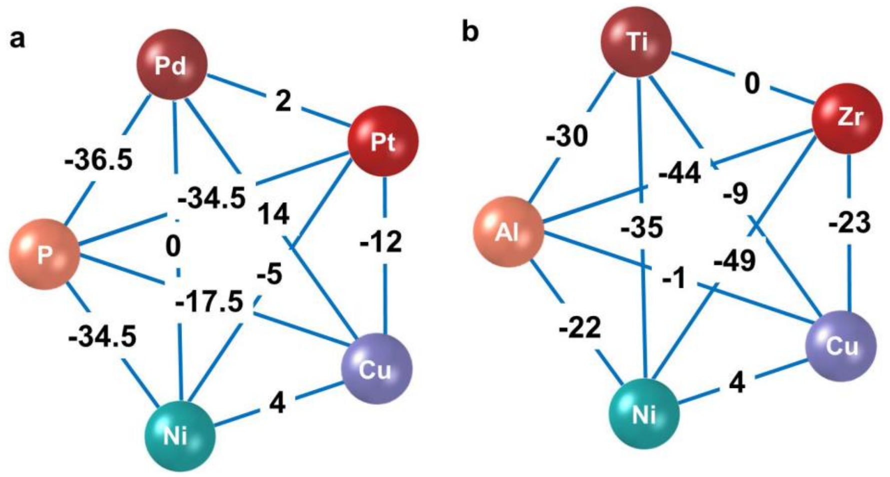

Supplementary Figure 1 | The value of ΔH^{mix} (kJ mol^{-1}) for the constituting atomic pairs. (a) The value of ΔH^{mix} (kJ mol^{-1}) for the elements in Pd(Pt)CuNiP system and **(b)** The value of ΔH^{mix} (kJ mol^{-1}) for the elements in Ti(Zr)CuNiAl system.

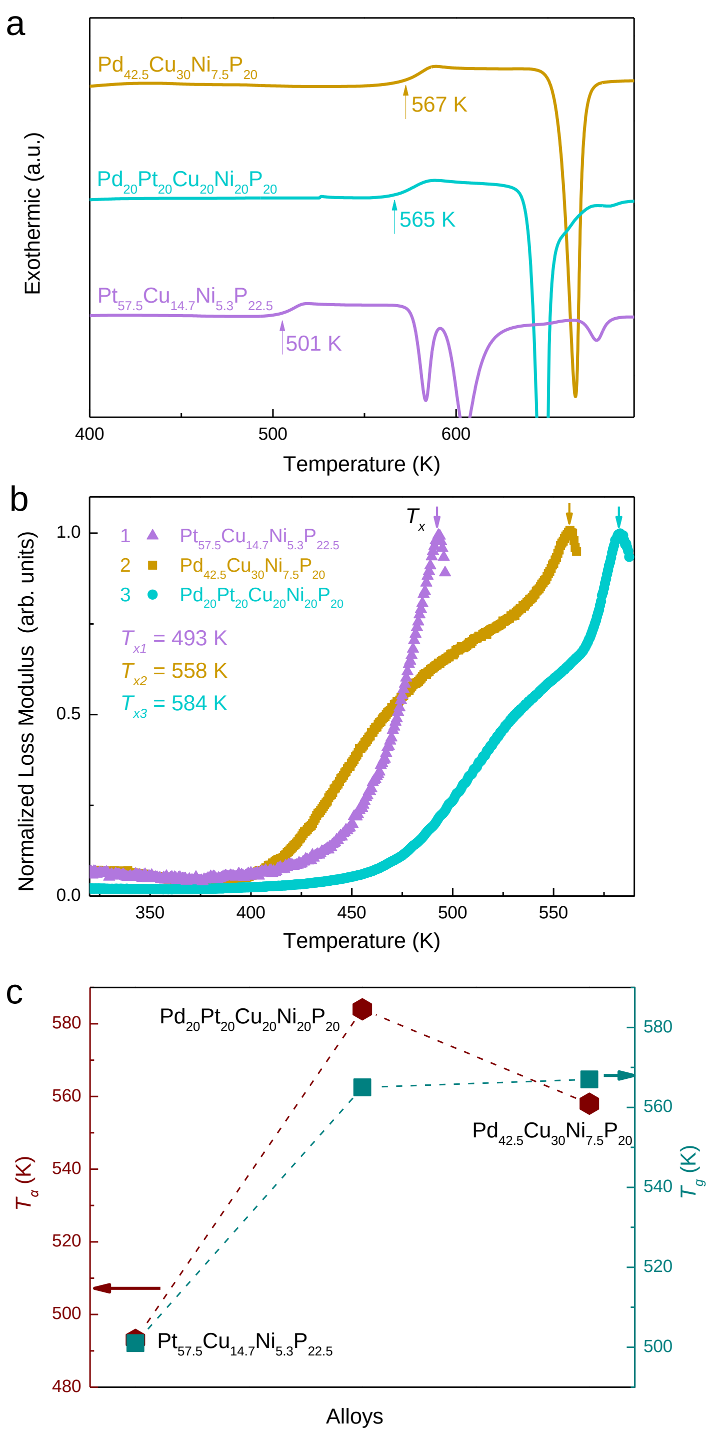

Supplementary Figure 2 | Thermodynamic and dynamical behaviors for Pd(Pt)CuNiP system. (a) DSC traces of Pd(Pt)CuNiP system with a heating rate of 0.33 K s^{-1}. **(b)** Temperature dependences loss modulus of Pd(Pt)CuNiP system at 1Hz with a constant heating rate of 0.05 K s^{-1}, normalized by the maximum peak value. **(c)** Plots of calorimetric T_g and dynamical T_α for Pd(Pt)CuNiP system.

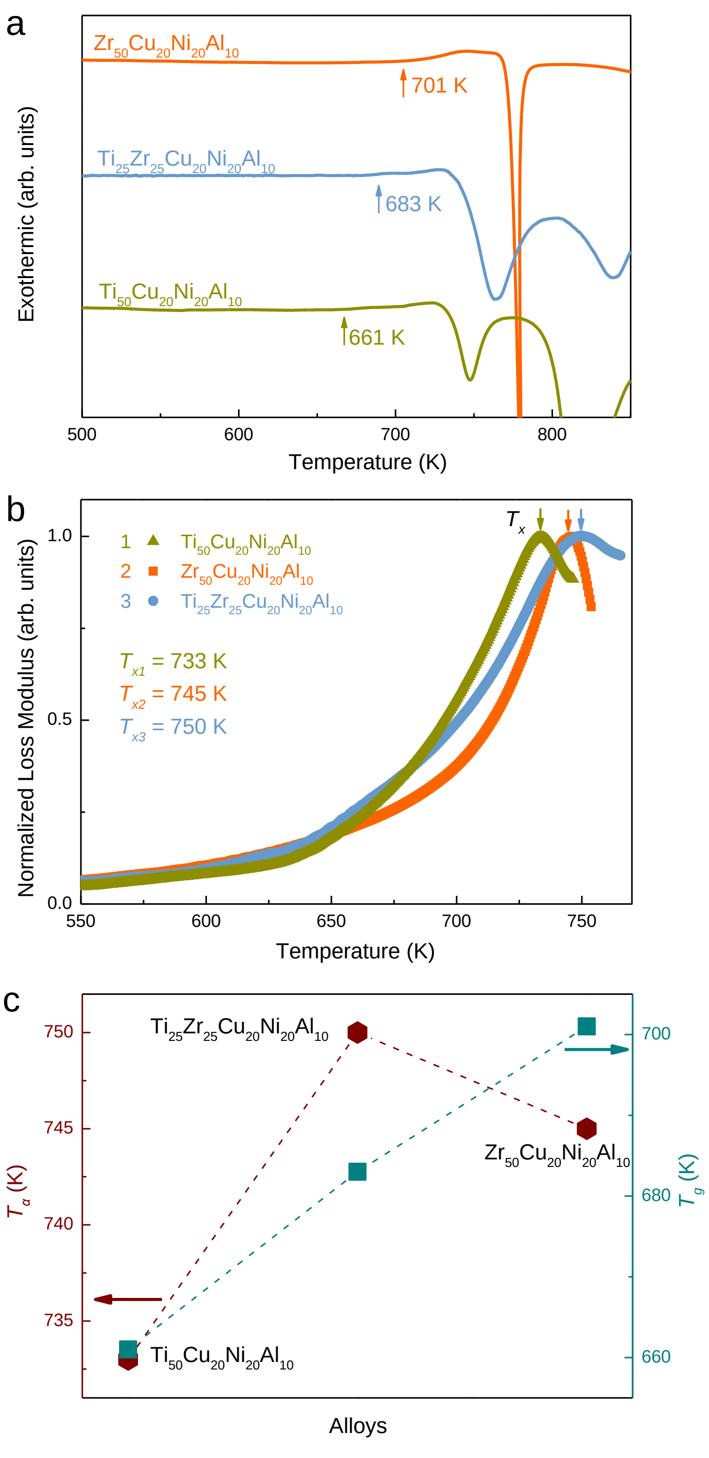

Supplementary Figure 3 | Thermodynamic and dynamical behaviors for Ti(Zr)CuNiAl system. (a) DSC traces of Ti(Zr)CuNiAl system with a heating rate of 0.33 K s^{-1}. **(b)** Temperature dependences loss modulus of Ti(Zr)CuNiAl system at 1Hz with a constant heating rate of 0.05 K s^{-1}, normalized by the maximum peak value. **(c)** Plots of calorimetric T_g and dynamical T_α for Ti(Zr)CuNiAl system.

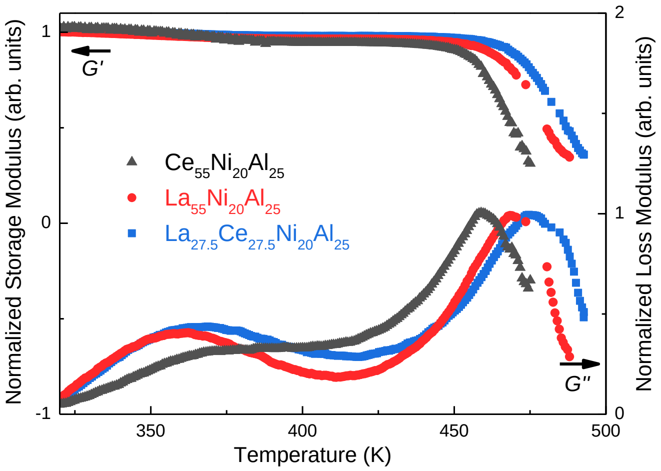

Supplementary Figure 4 | Dynamical behaviors for La(Ce)NiAl system. Temperature dependences loss modulus (G'') and the storage modulus (G') at 1Hz with a constant heating rate of 0.05 K s^{-1}. G'' is normalized by the maximum peak value, G' is normalized by the initial value.

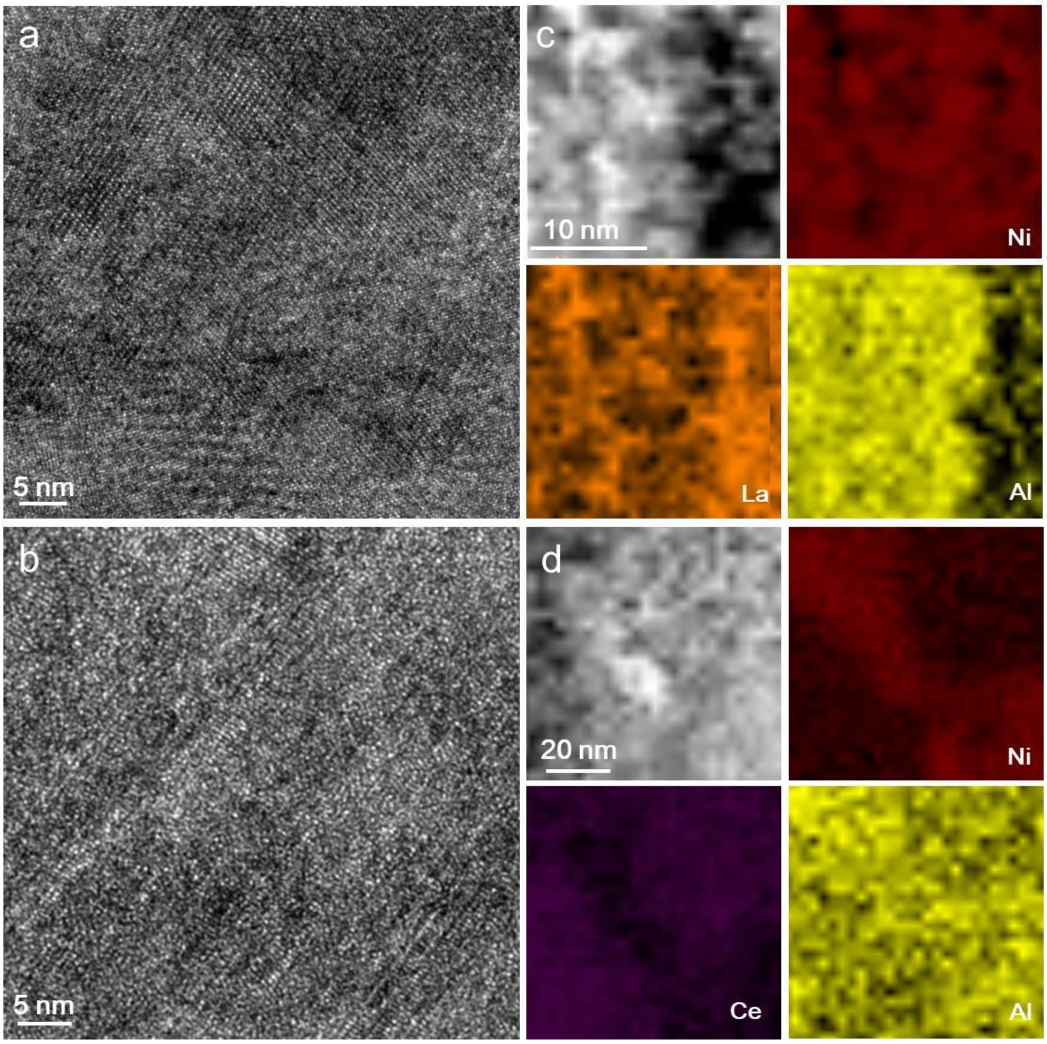

Supplementary Figure 5 | Atomic structure information of crystallized MGs. (a) and (b) HRTEM images of the crystallization regions for $La_{55}Ni_{20}Al_{25}$ and $Ce_{55}Ni_{20}Al_{25}$, respectively. **(c) and (d)** Corresponding EDS mapping after crystallization for $La_{55}Ni_{20}Al_{25}$ and $Ce_{55}Ni_{20}Al_{25}$, respectively.

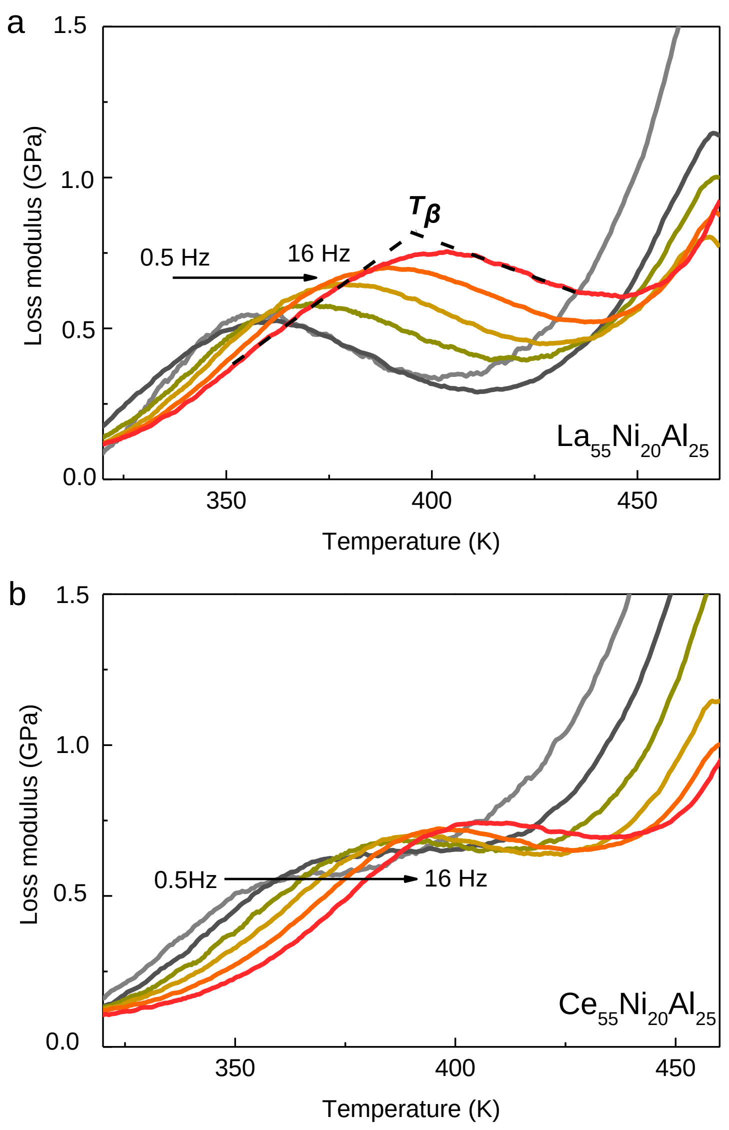

Supplementary Figure 6 | Dynamical *β*-relaxation behaviors of MGs. (a) and **(b)** Frequency-dependence of *β*-relaxation evolution of $La_{55}Ni_{20}Al_{25}$ and $Ce_{55}Ni_{20}Al_{25}$ respectively. The used frequencies are ranging from 0.5 to 16 Hz. Value of T_β is determined by the intersection of tangent lines of the *β*-relaxation peak which is marked in **(a)**.

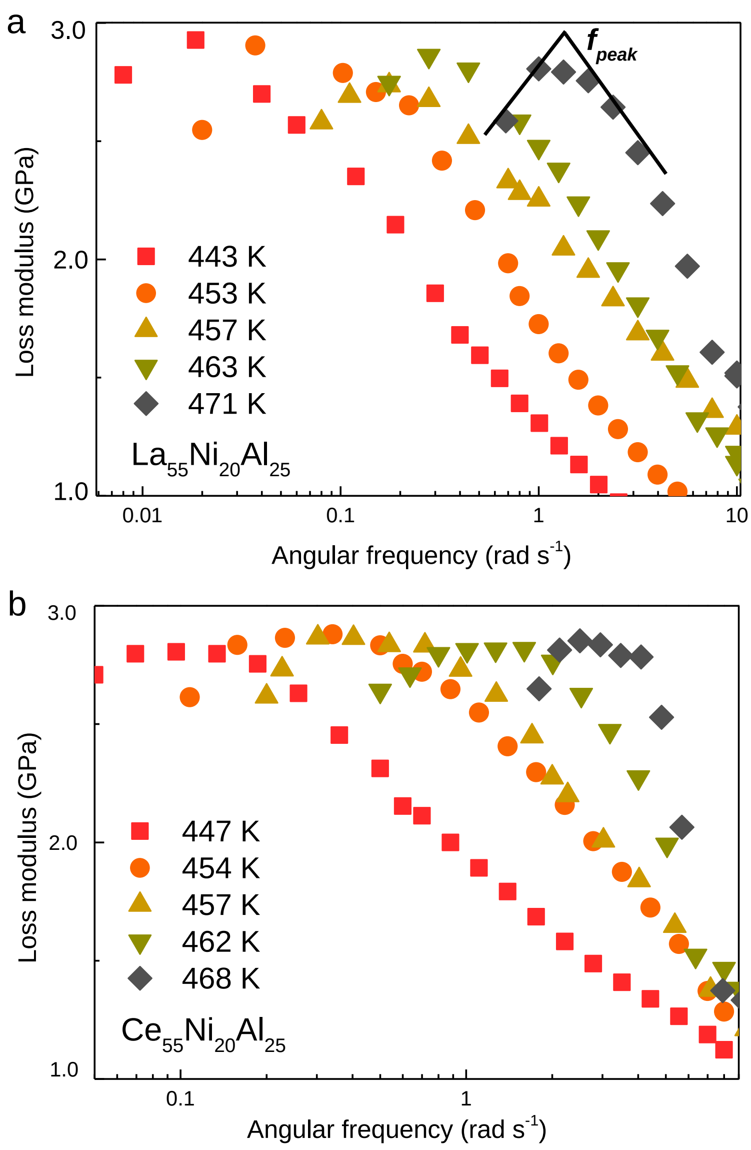

Supplementary Figure 7 | Dynamical α-relaxation behaviors of MGs. (a) and **(b)** Temperature-dependence of the evolutions of α-relaxation for $La_{55}Ni_{20}Al_{25}$ and $Ce_{55}Ni_{20}Al_{25}$ respectively. Value of f_{peak} is determined by the intersection of tangent lines of the α-relaxation peak which is marked in **(a)**.

Supplementary Table 1 | Average characteristic sizes of La(Ce)NiAl alloy system in different statuses.

**Average size**	**As cast status (nm)**	**After annealing (nm)**
$La_{55}Ni_{25}Al_{20}$	2.491 ± 0.297	30 ± 8
$Ce_{55}Ni_{25}Al_{20}$	2.380 ± 0.248	30 ± 10
$La_{27.5}Ce_{27.5}Ni_{25}Al_{20}$	1.851 ± 0.206	8 ± 1.7
